# Supplementary figures and images for: iPRESTO: Automated discovery of biosynthetic sub-clusters linked to specific natural product substructures
Source: PLoS Comput Biol. 2023 Feb 9;19(2):e1010462. doi: 10.1371/journal.pcbi.1010462 (PMC9946207; doi:10.1371/journal.pcbi.1010462)

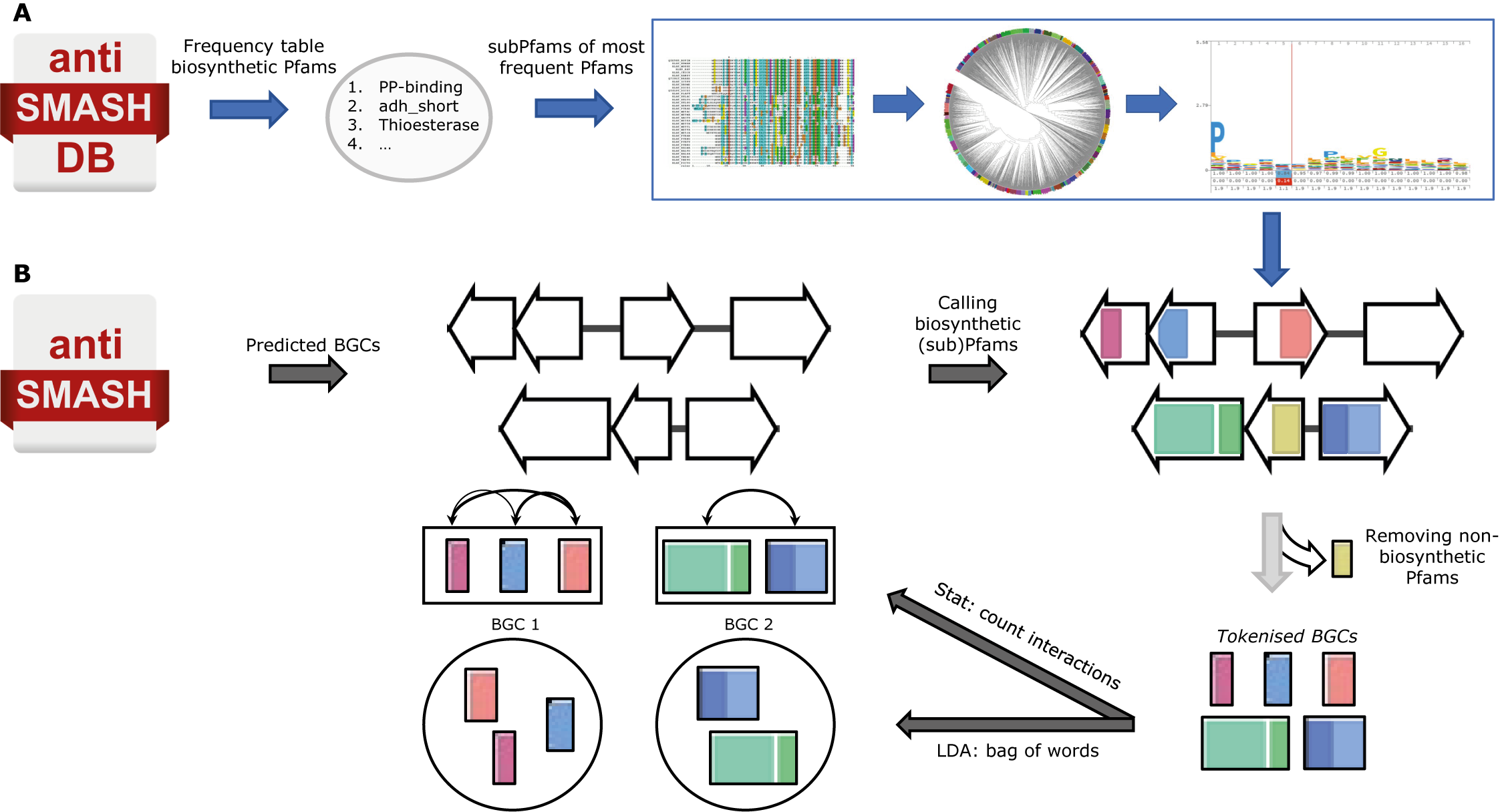

Supplement: S1 Fig — (A) subPfams are constructed for the 112 most frequent Pfam domains in the antiSMASH-DB by dividing the multiple sequence alignment of a Pfam into clades and converting each clade into a new pHMM. (B) The BGCs predicted by antiSMASH are tokenised by detecting (sub)Pfams in each gene, where non-biosynthetic Pfams are removed. After tokenising the BGCs, sub-cluster can be predicted with the statistical method (Stat), where the tokenised genes are represented in their original order, or by LDA, which assumes a bag of words model where original gene order is not considered. (TIF) [file pcbi.1010462.s002.tif]

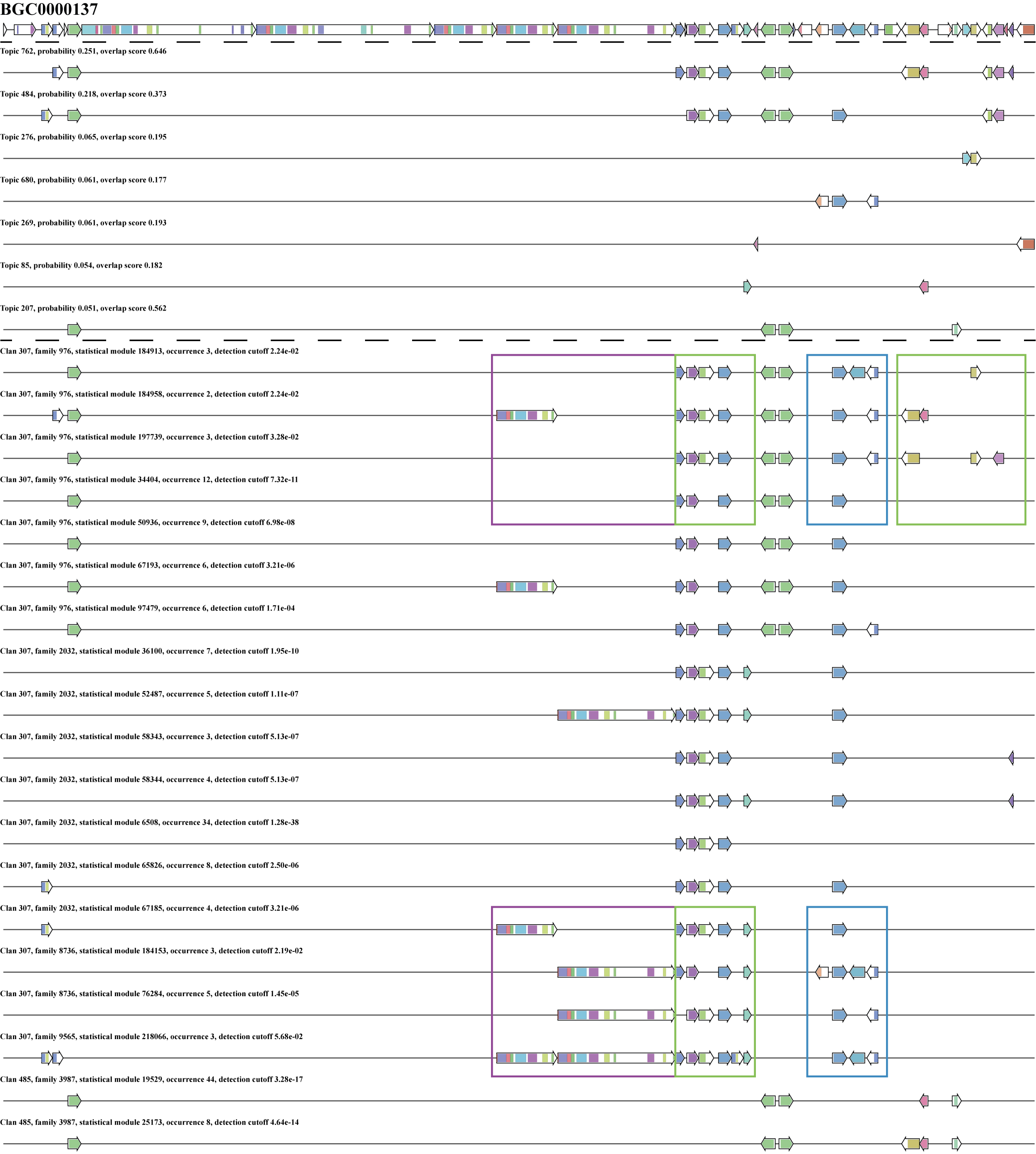

Supplement: S2 Fig — Only around 25% of the PRESTO-STAT sub-clusters are shown. Each gene is depicted as a token, where all (sub)Pfam domains are coloured. The visualisation of the BGC, the PRESTO-TOP and PRESTO-STAT output are separated by a dashed line, respectively. All PRESTO-STAT sub-clusters clearly exhibit a nested structure, where all combinations of genes in an actual sub-cluster are predicted as individual sub-clusters. The PRESTO-STAT sub-clusters shown here are also examples of noisy sub-clusters comprised of combinations of genes from different actual sub-clusters, like predicted PRESTO-STAT sub-clusters that are combinations of genes responsible for the biosynthesis of AHBA (green), sugars (blue) and the polyketide scaffold (purple). (TIF) [file pcbi.1010462.s003.tif]

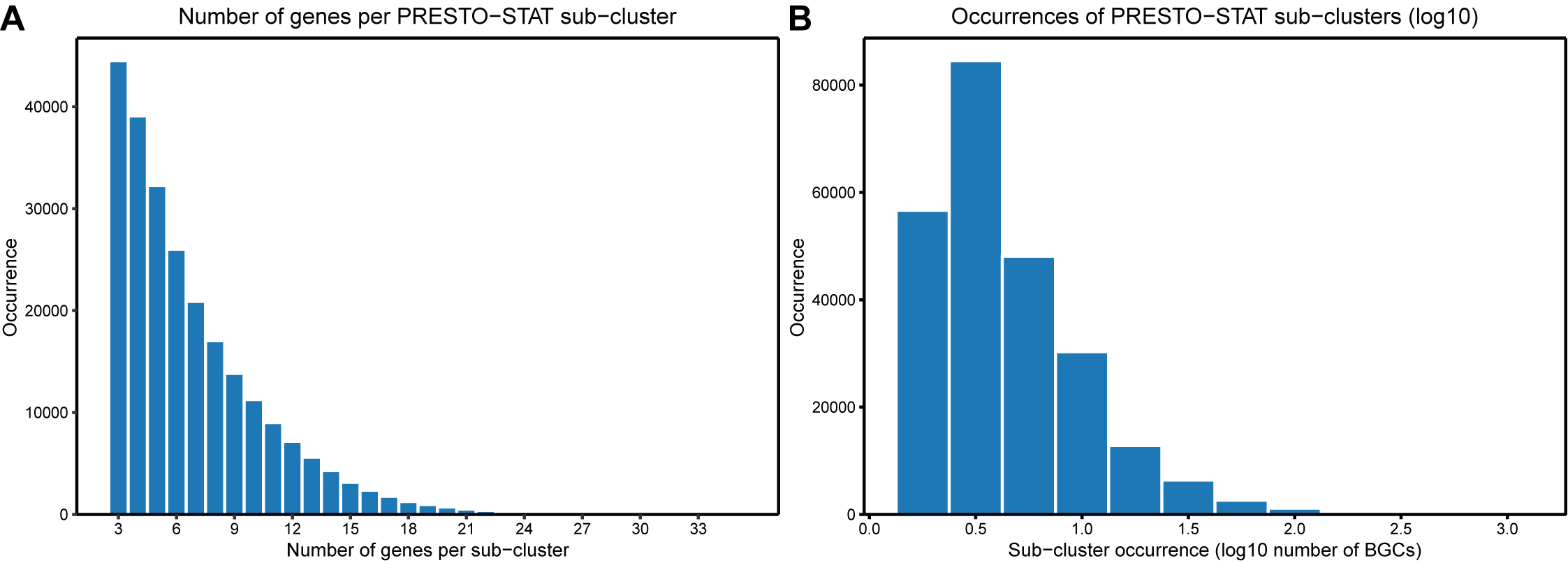

Supplement: S3 Fig — (A) The distribution of the number of genes per PRESTO-STAT sub-cluster in the antiSMASH-DB dataset. (B) The distribution of the log10 transformed PRESTO-STAT sub-cluster occurrences in the antiSMASH-DB dataset. (TIF) [file pcbi.1010462.s004.tif]

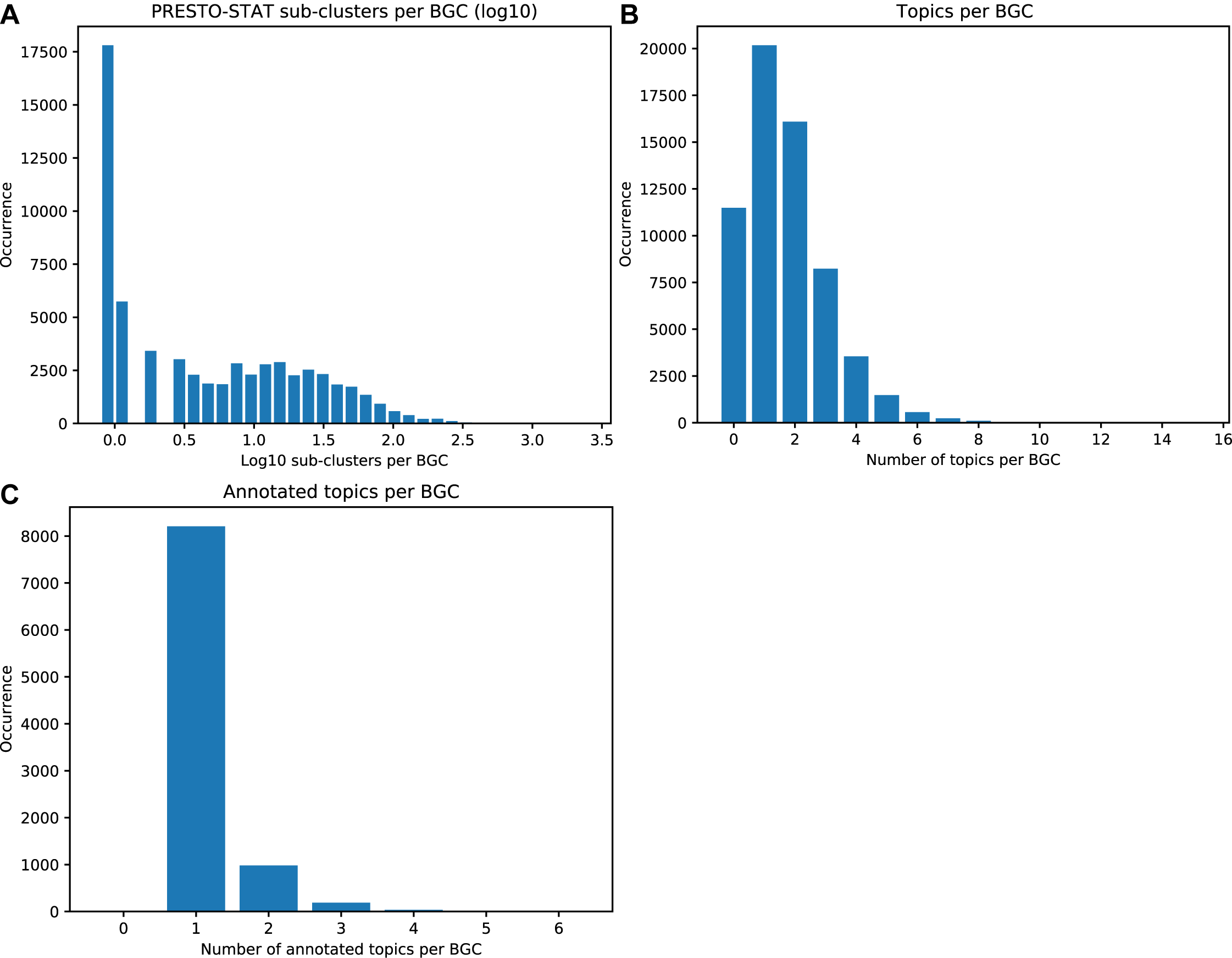

Supplement: S4 Fig — (A) Distribution of the log10 transformed number of PRESTO-STAT sub-clusters per BGC in the non-redundant antiSMASH-DB dataset, where the bin with the seemingly negative value represents BGCs without any PRESTO-STAT sub-cluster. (B) The number of topics or sub-cluster motifs per BGC in the non-redundant antiSMASH-DB dataset, not counting sub-clusters of length one as these are almost definitely noise (see Methods). (C) All BGCs with at least one annotated sub-cluster motif grouped by how many annotated sub-cluster motifs they have. In total there are 9,425 putative BGCs with at least one annotated sub-cluster motif, and 350 MIBiG BGCs. (TIF) [file pcbi.1010462.s005.tif]

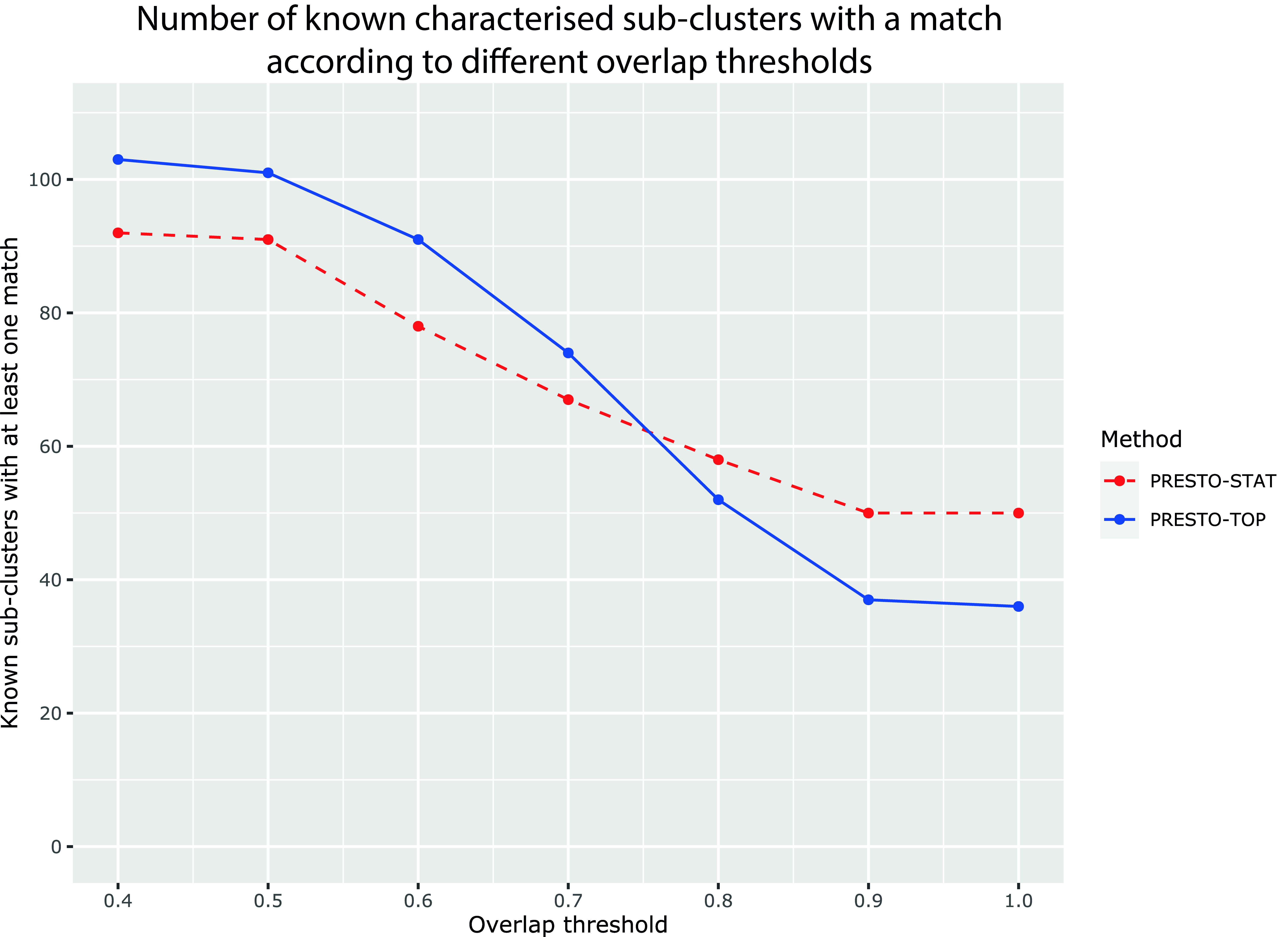

Supplement: S5 Fig — Overlap between predicted SubClusterBlast sub-clusters and output of both sub-cluster prediction methods applied on the antiSMASH-DB dataset according to different overlap cut-offs. The overlap expresses the fraction of genes from the original SubClusterBlast sub-cluster that is found in the iPRESTO-predicted sub-cluster. We considered an overlap of 0.6 sufficient for having predicted a sub-cluster (see Supplementary methods in S1 Text). (TIF) [file pcbi.1010462.s006.tif]

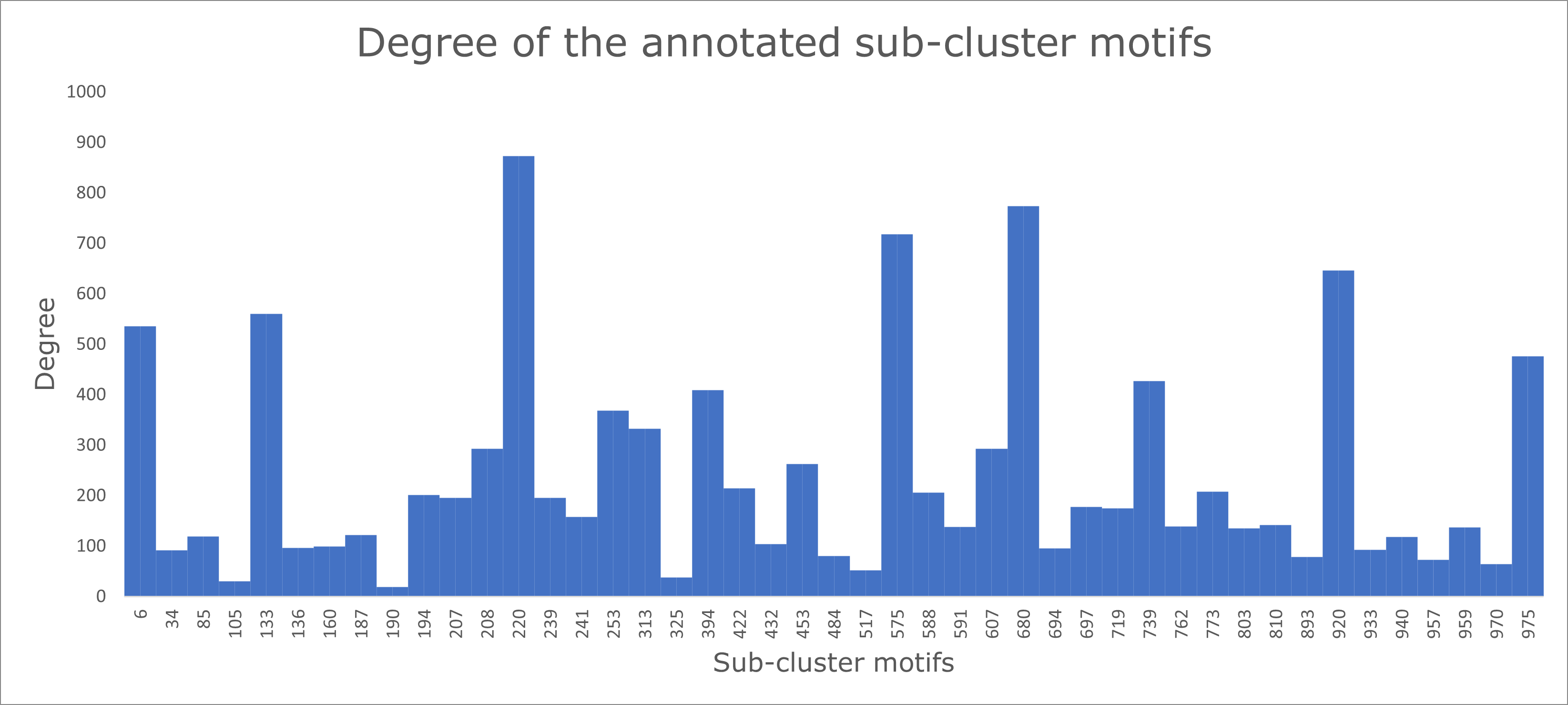

Supplement: S6 Fig — (TIF) [file pcbi.1010462.s007.tif]

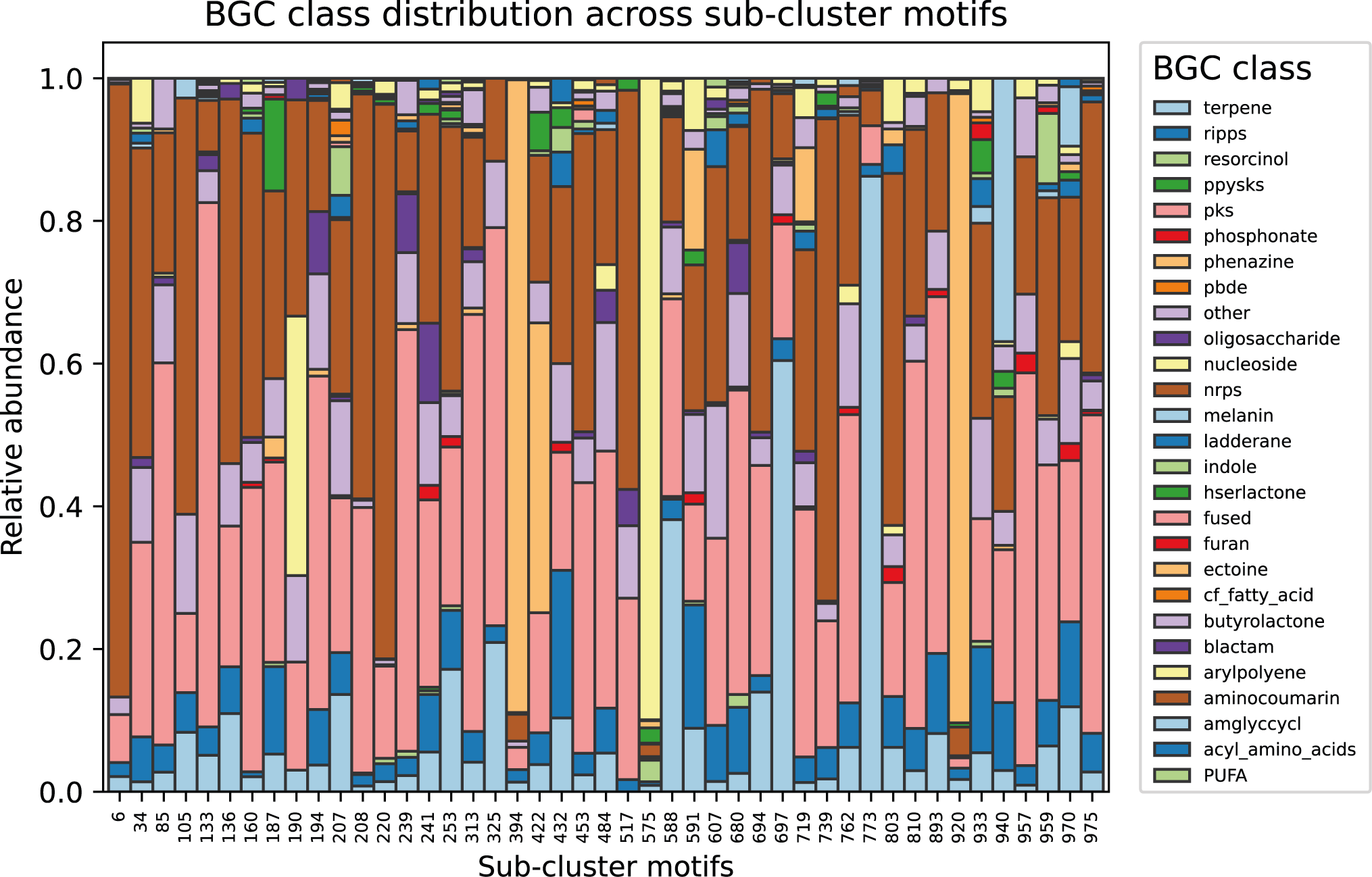

Supplement: S7 Fig — Relative abundance of antiSMASH classes when querying the non-redundant antiSMASH-DB dataset on the 45 annotated sub-cluster motifs. Matches of length 1 are ignored and hybrid class BGCs are counted for all classes they contain. RIPPs classes are grouped together. (TIF) [file pcbi.1010462.s008.tif]

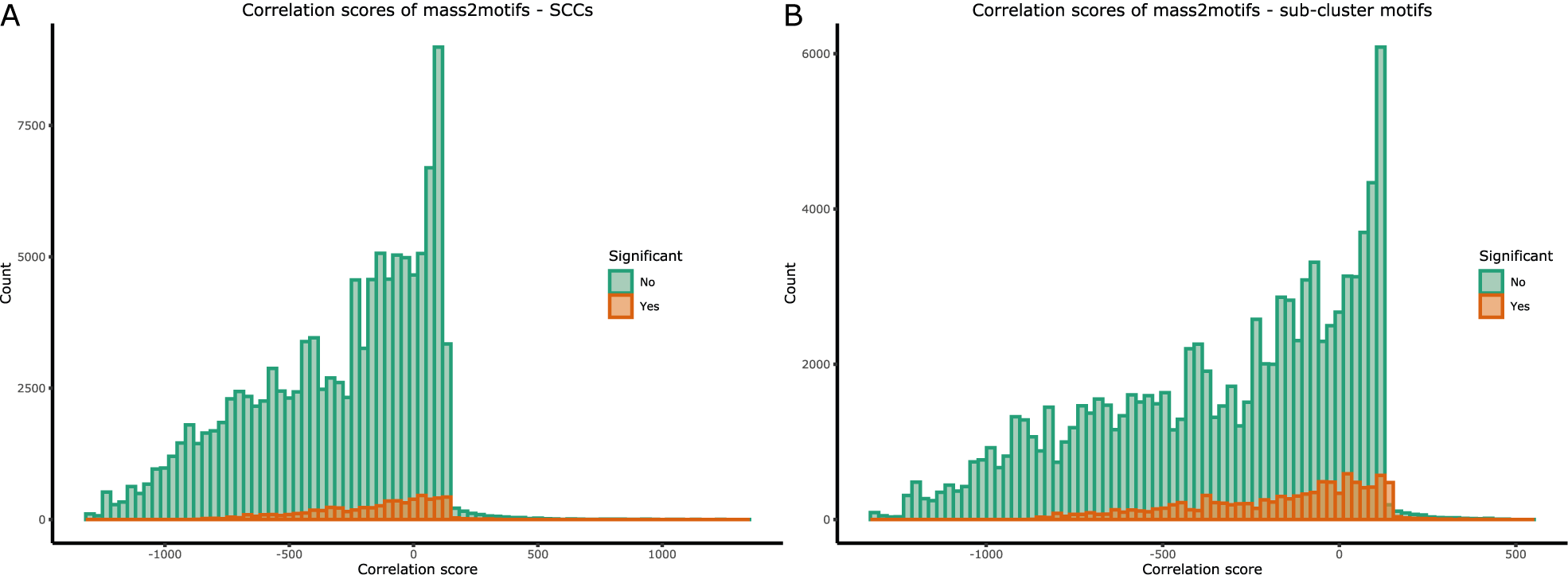

Supplement: S8 Fig — (A) Correlation scores between Mass2Motifs and SCCs. (B) Correlation scores between Mass2Motifs and sub-cluster motifs. In both panels the significant pairs are highlighted. (TIF) [file pcbi.1010462.s009.tif]

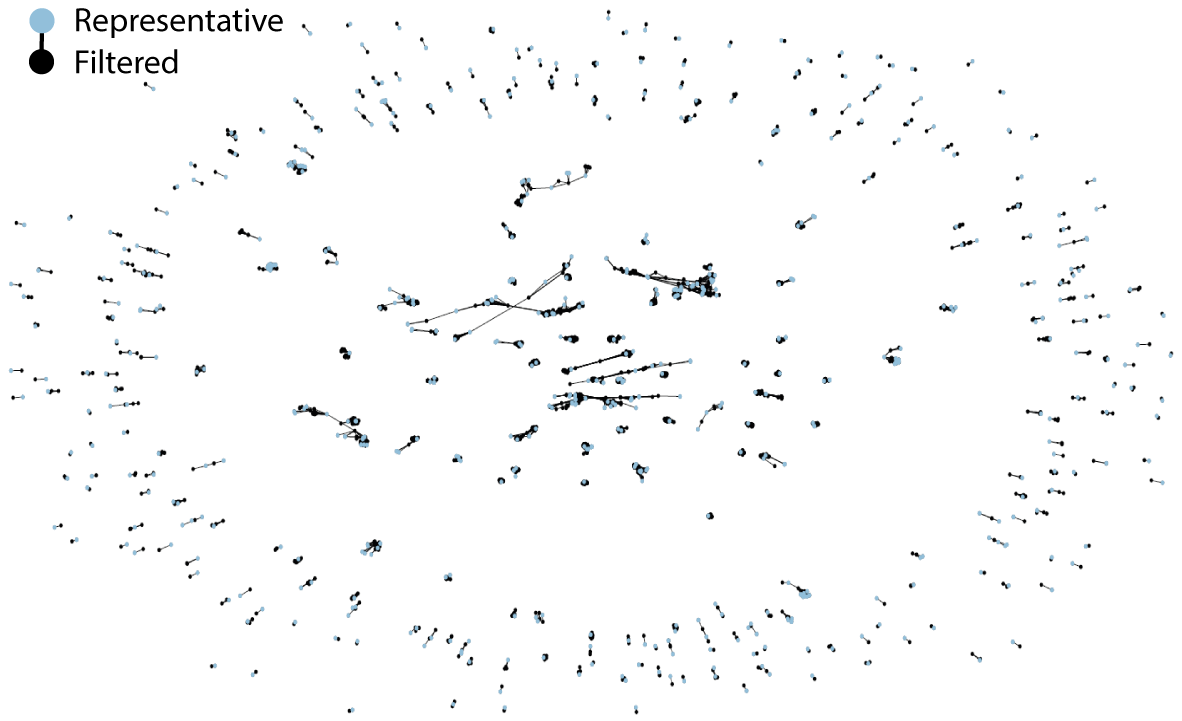

Supplement: S9 Fig — Each node represents a BGC and an edge represents an adjacency index (AI) of 0.95 or higher. In blue are the BGCs chosen as representatives, while BGCs that are filtered out are shown in black. We show the small dataset here as it was difficult to visualize this process for the antiSMASH-DB dataset. (TIF) [file pcbi.1010462.s010.tif]

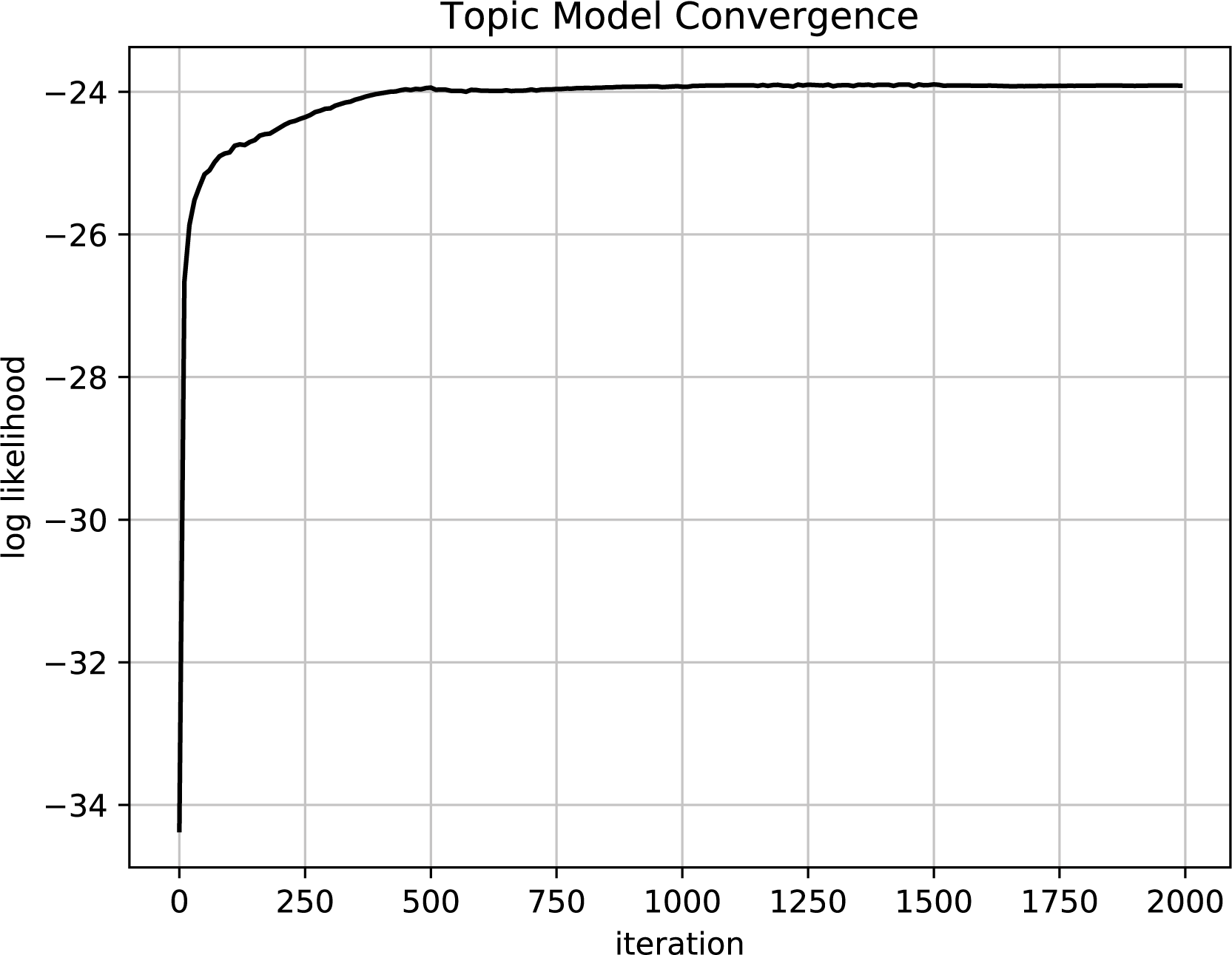

Supplement: S10 Fig — Convergence of the log-likelihood of an LDA model with 1,000 topics/sub-cluster motifs trained on the non-redundant 60,028 BGCs from the antiSMASH-DB dataset, which also contains the Streptomyces/Salinispora dataset and the MIBiG database, using 2,000 iterations of chunk size 3,000. Log-likelihood based on 28 held out BGCs. (TIF) [file pcbi.1010462.s011.tif]

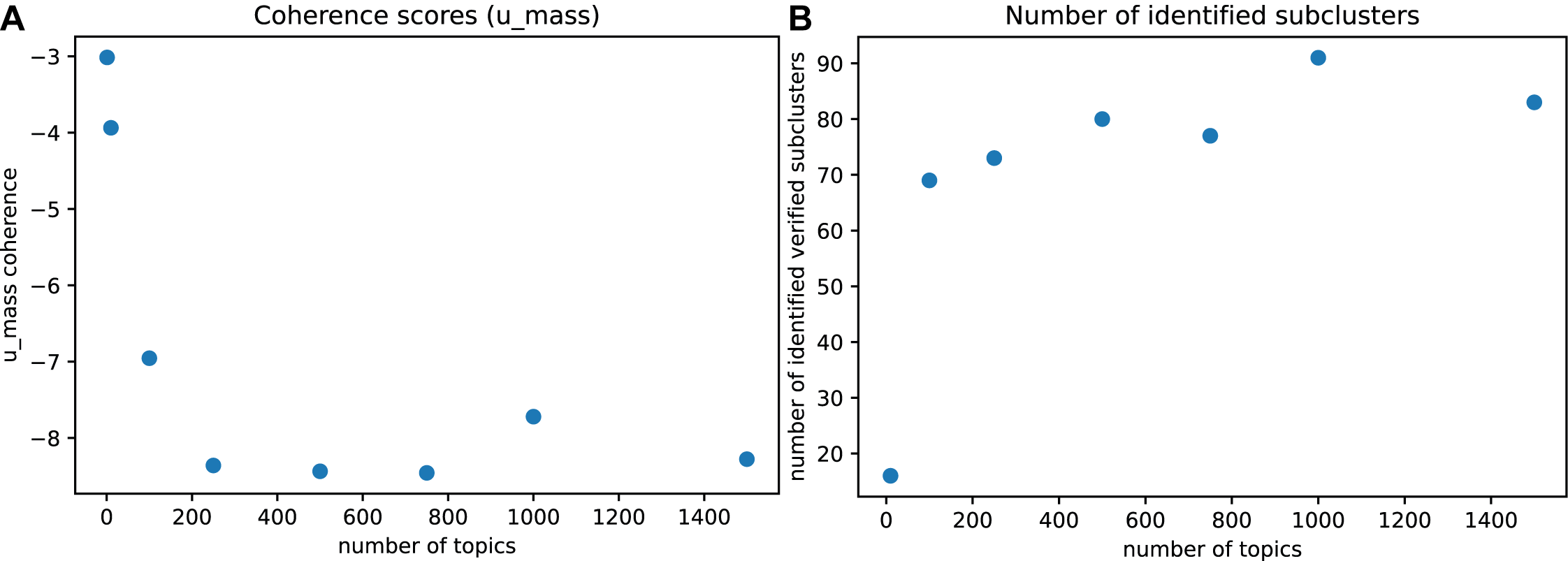

Supplement: S11 Fig — (A) Coherence scores of different LDA models trained using PRESTO-TOP on the non-redundant antiSMASH-DB dataset with different number of topics. (B) Number of validated SubClusterBlast sub-clusters found with different LDA models trained using PRESTO-TOP on the non-redundant antiSMASH-DB dataset with different number of topics. (TIF) [file pcbi.1010462.s012.tif]
