# Supplementary material for: iPRESTO: Automated discovery of biosynthetic sub-clusters linked to specific natural product substructures
Source: PLoS Comput Biol. 2023 Feb 9;19(2):e1010462. doi: 10.1371/journal.pcbi.1010462 (PMC9946207; doi:10.1371/journal.pcbi.1010462)
Supplement: Attachment — Submitted filename: iPRESTO_Response_to_Reviewers_20230109.pdf [file pcbi.1010462.s017.pdf]

Rebuttal letter iPRESTO: automated discovery of biosynthetic sub-clusters linked to specific natural product substructures

We thank the reviewers and editor for their generally very positive comments on our submitted manuscript. We have carefully consider the remaining points and revised our contribution as such. Please find our point-by-point response below. Author comments are written in blue. A tracked changes version of the manuscript highlights all the changes made, of which the most important are also copied below.

Reviewer #1: The identification of subclusters within predicted BGCs is highly useful for both attempting to link a cryptic BGC to a proposed molecule or for attempts to predict encoded structure in the process of discovery. I believe iPRESTO will make an invaluable contribution to the Antismash framework. The manuscript is well written and I believe it should be accepted for publication. I only have two small comments that I believe will improve the manuscript and the ability of readers to better utilize this study.

Thanks for your positive words and your valuable suggestions and we agree that future further integration within the antiSMASH framework would facilitate natural product research.

- Only 45 subclusters were annotated but I believe the remaining unannotated subclusters should be provided in the excel spreadsheet, perhaps in a different tab. Information on these remaining subclusters may help other researchers with their predictions or may even be annotated by other studies in the future.

Thanks for this suggestion. Following your remark, we have now added an additional tab to the excel sheet containing information about each sub-cluster motif. We also added more files to the supplemental data hosted on Zenodo (https://doi.org/10.5281/zenodo.6953656) such that users can find more details if they want to. The most important of which is "mibig_gbk_2.0_clusterfile_ipresto_output_visualisation.html" that contains a visualization of all MIBiG BGCs queried to the collection of sub-clusters generated in this project. akin to S2 Fig.

We clarified this in the manuscript as follows:

"S1 File. Excel file containing the current information about the generated sub-cluster motifs. Sheet one includes annotations for the annotated sub-cluster motifs and sheet two contains metadata for all sub-cluster motifs such as biosynthetic classes and taxonomic assignments of BGCs that are associated with them." (Page 13)

"Additional information about the sub-cluster motifs and other relevant data can be found at https://doi.org/10.5281/zenodo.6953656. This includes a visualisation of all MIBiG BGCs queried to the set of sub-clusters generated in this study." (Page 13)

- Whilst the authors sometimes say that only inferences are made – I would like to urge the authors to remember that these are in fact only bioinformatic predictions and that direct evidence is lacking, particularly in the earlier parts of the manuscript. Whilst it is stated sometimes, I would like to ask the authors to go through the manuscript and check their language to ensure this aspect is clear.

We agree that it is important to realize that iPRESTO makes predictions and to ensure that the readers keep this in mind, we have adjusted our wording as follows: we replaced "detection" by "prediction" at various instances and only kept "detection" when discussing experimentally validated sub-clusters (throughout the document).

Reviewer #2: Louwen et al. present iPRESTO, a tool which can explore a database of biosynthetic gene clusters (BGCs) to extract common subcluters. They adapt LDA (latent Dirichlet allocation) for this problem, which improves over the previous state-of-the-art. They show that they can reproduce some known findings and find some suggestive novel assignments for certain metabolites.

Overall, the work is well executed and the value of the approach is well demonstrated. I have only minor comments on how the work is presented.

Thanks for your positive words and your valuable suggestions.

Minor Comments

#1 The paper is framed as a tool that analyses a collection of BGCs, but to me it seems that most of the value is in the database that the authors explore. On the git repository, it seems that it is possible for a user to map their BCGs into the motifs that the authors have discovered ("Querying existing sub-cluster models"). I estimate that this is much more useful than running the subcluster discovery from scratch and I suggest mentioning it more prominently in the manuscript. The authors do note that this will be included in antiSMASH in the future, but if the functionality is already available on the command line tool, it could be mentioned in the manuscript.

We agree that the main use of iPRESTO is to query BGCs to existing sub-cluster models that are (partially) annotated, like the ones we detected in this study. iPRESTO is indeed already available as a command line tool. We have now clarified this in several sections:

Introduction (Page 4, top): “iPRESTO is available as a command-line tool at https://git.wageningenur.nl/bioinformatics/iPRESTO/. We anticipate that the main use of iPRESTO for the genome-mining community is to query BGCs to the annotated sub-cluster motifs that we made available, and, in doing so, predicting one or more substructures in the biosynthetic products of those BGCs. To make our current analyses most useful, we provide both annotated sub-clusters as well as the remaining unannotated sub-clusters together with information such as the biosynthetic classes and taxonomic assignments of the BGCs that are associated with them, so that they can continue to be explored. With new training data, it is of course also possible to generate novel sub-cluster models with iPRESTO.”

Results (Page 4, bottom): “iPRESTO is readily usable as a command-line tool for anyone who wants to detect sub-clusters in their own datasets, by querying BGCs to the collection of sub-clusters we detected and partially annotated in this study. It is also possible to use iPRESTO for detecting new sub-cluster models from scratch using new training data.”

Conclusion and future perspectives (Page 8, bottom): “This study introduces the iPRESTO concept and makes it available as a command-line tool which can query BGCs to the set of partially annotated sub-clusters generated in this study, as well as to train new sub-cluster models. We plan to include iPRESTO in one of the future releases of antiSMASH, so the collection of sub-clusters we generated can be used more easily to detect and visualize them in antiSMASH-predicted BGCs.”

#2 PRESTO-STAT should be introduced in a way that is understandable even for readers that are not familiar with the prior method on which it is based.

Thank you for your remark. We provided additional explanations lines XXX:

Introduction (Page 3, bottom): “PRESTO-STAT uses the same approach as the method by Del Carratore et al. [10] to find sub-clusters based on genes that co-occur in a statistically significant fashion across a collection of BGCs. We further developed the method by removing part of the nested sub-clusters and collapsing similar sub-clusters into families and clans. PRESTO-TOP is a novel method for sub-cluster prediction based on Latent Dirichlet Allocation (LDA) that learns a set of sub-cluster motifs from a collection of BGCs.”

#3 The motivation for why it is a positive thing that many nested sub-clusters are avoided by PRESTO-STAT/PRESTO-TOP should be explained better. IIUC, the problem with previous methods is that it reports too many overlapping/redundant nested sub-clusters. This should be made explicit early.

Such nested sub-clusters are a problem as it confounds the results and makes it even more difficult to find actual biologically meaningful sub-clusters as nested combinations of biological sub-clusters are highly present in the output. We have now clarified this in the Introduction (Page 3, middle):

“Apart from (artificially) inflating the number of sub-clusters, nested structures also make it more difficult to find actual biologically meaningful sub-clusters as a result of nested combinations of biological sub-clusters.”

#4 Line 164: This is unclear to me. If I understand it correctly, the problem is to query a BCG for whether it contains (or approximately contains) any known sub-clusters. Even a fairly naive index would probably work because the number of subclusters that each Pfam domain only participates in is not that large. Perhaps I have misunderstood what was meant.

The reviewer is right: indeed, checking whether a BGC contains a PRESTO-STAT sub-cluster is rather fast. However, when querying unknown BGCs that are less likely to exactly resemble detected sub-clusters from the training data, one would like to check if a BGC of interest approximately contains a sub-cluster. This would quickly become more time consuming when combinatorially checking all possible ways of matching a sub-cluster minus, for example, two genes that are missing. Our point indeed related to the time scale of the operation, not to the difficulty. We therefore rephrased the referred line into:

“Additionally, it would quickly become very time-consuming to query a BGC using the statistical sub-clusters while also allowing inexact matching”

Line 469: I do not understand the sentence "For a sub-cluster to be considered it needs to consist of more than one gene, for which we set a cut-off of 1.1 on the summed feature probabilities." Which feature probability are being summed and how can their sum exceed 1.0?

We here meant the probability of a tokenised gene (feature) being assigned to the motif. This ranges from 0 to 1, or, for example, from 0 to 3 if there are three of the same genes in a BGC. So if we sum these “gene-to-motif” probabilities, we can set a threshold on this to make sure that a sub-cluster consists of more than one gene. We have now clarified this in the manuscript in the Methods section on Page 11 , bottom:

"When a group of genes from a BGC match to a sub-cluster motif, each gene is assigned a gene-to-motif probability describing how well it fits in the sub-cluster motif, for which we set a cut-off of 0.3. To consider the matching group of genes a sub-cluster, it needs to consist of more than one gene. We therefore set a cut-off of 1.1 on the summed feature gene-to-motif probabilities.”

Line 183: Figure reference is incomplete, should probably be 2C.

Thank you for noticing. We adjusted the reference to 2C.
